# The more, the better? Learning rate and self-pacing in neurofeedback enhance cognitive performance in healthy adults

**DOI:** 10.3389/fnhum.2023.1077039

**Published:** 2023-01-17

**Authors:** Sinan Uslu, Claus Vögele

**Affiliations:** Department of Behavioural and Cognitive Sciences, University of Luxembourg, Esch-sur-Alzette, Luxembourg

**Keywords:** neurofeedback, individual upper alpha, learning rate, self-paced training, electroencephalography (EEG), cognitive performance

## Abstract

Real time electroencephalogram (EEG) based neurofeedback has been shown to be effective in regulating brain activity, thereby modifying cognitive performance and behavior. Nevertheless, individual variations in neurofeedback learning rates limit the overall efficacy of EEG based neurofeedback. In the present study we investigated the effects of learning rate and control over training realized by self-pacing on cognitive performance and electrocortical activity. Using a double-blind design, we randomly allocated 60 participants to either individual upper alpha (IUA) or sham neurofeedback and subsequently to self- or externally paced training. Participants receiving IUA neurofeedback improved their IUA activity more than participants receiving sham neurofeedback. Furthermore, the learning rate predicted enhancements in resting-state activity and mental rotation ability. The direction of this linear relationship depended on the neurofeedback condition being positive for IUA and negative for sham neurofeedback. Finally, self-paced training increased higher-level cognitive skills more than externally paced training. These results underpin the important role of learning rate in enhancing both resting-state activity and cognitive performance. Our design allowed us to differentiate the effect of learning rate between neurofeedback conditions, and to demonstrate the positive effect of self-paced training on cognitive performance in IUA neurofeedback.

## 1. Introduction

Neurofeedback is a neurocognitive intervention which enables users to regulate their brain activity *via* a three-step iterative loop: (1) measuring brain activity, (2) processing it and (3) feeding it back to the user. The learning of this regulation relies on the principles of operant conditioning by providing rewarding feedback whenever the user successfully regulated the brain activity and, furthermore, it is conceptualized in the context of control-theoretical models involving neurophysiological processes and the dual-process theory including automated processes (for a review see Enriquez-Geppert et al., 2017). This reinforcement and increase in successful regulation are finally intended to change cognitive and behavioral outcomes, which are related to the targeted brain activity (Enriquez-Geppert et al., 2017). In electroencephalography (EEG) based neurofeedback, training protocols typically target the regulation of frequency bands by decomposing the EEG signal during neurofeedback training.

Many studies have demonstrated the efficacy of neurofeedback to regulate brain activity and, thereby, to modify cognitive performance and behavior in both clinical (Garcia Pimenta et al., 2021) and healthy samples (Nan et al., 2012; Escolano et al., 2014; Gruzelier, 2014; Navarro Gil et al., 2018). In clinical populations, neurofeedback has been shown to enable patients to regulate their brain activity and thereby to influence their symptoms. For example, in attention deficit hyperactivity disorder (ADHD) the treatment efficacy of neurofeedback was comparable to those of other intervention types and further improved when personalized by selecting the brain activity feature to modulate based on EEG characteristics measured prior to neurofeedback (for a review see Garcia Pimenta et al., 2021). In their review the authors further discussed heightened reward sensitivity in children diagnosed with ADHD as a non-specific factor contributing to the training effect on clinical outcomes (Garcia Pimenta et al., 2021). While neurofeedback applications in clinical populations have focused on restoring cognitive and behavioral functionality, studies with non-clinical samples have concentrated on enhancing cognitive performance. Across varying training protocols, many studies could show cognitive improvements after regulating the alpha frequency range from the EEG spectrum (Gruzelier, 2014). For example, participants performing a single session of individual upper alpha (IUA) neurofeedback over occipitoparietal regions improved their IUA activity during training more than participants receiving sham neurofeedback (Escolano et al., 2014). Furthermore, participants receiving IUA neurofeedback also increased their higher-level cognitive skills measured with part B of the trail making task (TMT) more than participants receiving sham feedback, however, without differences in resting-state activity between the two groups. Even when participants underwent multiple sessions of IUA neurofeedback they did not show an increased IUA activity during a resting-state period compared to a waiting-list control group (Navarro Gil et al., 2018). In addition to enhancing cognitive performance, IUA neurofeedback also led to better short-term memory performance when compared to a non-neurofeedback waiting-list control group (Nan et al., 2012). The evidence from these studies also suggests that the increase in performance positively correlates with an increase in IUA activity (Nan et al., 2012; Navarro Gil et al., 2018).

Notably, the efficacy of neurofeedback varies across participants with some of them not increasing the targeted brain activity at all (Alkoby et al., 2018). While some researchers separate their analyses for participants who were successful in regulating their brain activity, hereafter referred to as “responders,” and for those who were not, hereafter referred to as “non-responders” (Hsueh et al., 2016; Autenrieth et al., 2020; Eschmann et al., 2022), other researchers have investigated the association between learning rate and outcome measures in a continuous manner (Nan et al., 2012; Navarro Gil et al., 2018; Naas et al., 2019). To explain these variations between participants, previous studies have assessed psychological (Thibault et al., 2016) and neurophysiological factors (Scheinost et al., 2014; Zhao et al., 2021) as predictors of improvements in behavioral and neurophysiological outcomes. In terms of neurophysiological factors, previous studies have used both measures of connectivity (Scheinost et al., 2014) and gray matter volume in resting-state fMRI as indicators of learning success (Zhao et al., 2021). For EEG based neurofeedback, researchers have demonstrated in a study in which all participants performed individual alpha band neurofeedback that resting-state relative alpha band power was positively correlated with the learning rate during training (Wan et al., 2014). Regarding psychological factors, researchers have investigated mental strategies participants used to regulate their brain activity (Kober et al., 2013), and the control beliefs participants had while dealing with technology (Witte et al., 2013). Participants reported different strategies after neurofeedback also depending on the targeted frequency band (Kober et al., 2013). For example, after sensory motor rhythm (SMR) based neurofeedback participants who reported not to have used a specific strategy enhanced their SMR activity more than participants reporting a strategy. Hence, up-regulation of SMR activity might depend on implicit associative learning mechanisms (Gruzelier, 2014). Some researchers have thus hypothesized that conscious efforts during neurofeedback interfere with non-conscious learning processes and may in turn decrease the learning rate (Witte et al., 2013). To summarize, both psychological and neurophysiological factors contributed to the overall efficacy of neurofeedback training and the findings suggest that those factors interact with the targeted feature of brain activity and, furthermore, depend on user characteristics.

In alpha neurofeedback the conscious pursuit of mental strategies positively influenced IUA band activity (Nan et al., 2012; Naas et al., 2019) and the individual adjustment of feedback levels yielded a wider range of feedback realized during training (Han et al., 2016). Participants were asked to pursue any mental strategy for a neurofeedback trial, but to stick to it during the trial (Nan et al., 2012). They were allowed to change strategies between trials. Reports gathered after neurofeedback indicated that positive strategies (e.g., thinking about friends) yielded increased activity in the IUA band compared to neutral (e.g., thinking about numbers) or negative strategies (e.g., thinking about accidents) (Nan et al., 2012). To control for non-specific factors, another study extended this methodology to a sham-controlled experiment (Naas et al., 2019). Interestingly, the learning rate was comparable between the groups indicating that appropriate mental strategies may be sufficient to enhance IUA activity. Nevertheless, hitherto no study has investigated the efficacy of mental strategies on IUA neurofeedback performance in a randomized controlled trial. Another approach to increase the efficacy of neurofeedback is focused on individually tailoring the training paradigm. For instance, the peak alpha frequency as a neurophysiological correlate of cognitive processes (Klimesch et al., 1993) enabled the individual adjustment of the targeted frequency range in neurofeedback training paradigms (Gruzelier, 2014). The mental rotation performance increased after stimulating participants with a transmagnetic stimulation frequency based on the individual alpha peak (Klimesch et al., 1993). In alpha neurofeedback studies, both the mental rotation task and TMT performance increased after the session (Hanslmayr et al., 2005; Escolano et al., 2014). Other parameters than the neurophysiological activity at resting-state which allow for individual adaptation include feedback thresholds (Han et al., 2016) and pacing of the training. Self-paced training would grant users more control over the distribution of their training time and allow them to explore mental strategies on their own pace.

The main goal of the current study was to extend previous research by estimating the effect of learning rate on resting-state IUA activity and cognitive performance after a single session of IUA neurofeedback and to investigate the efficacy of control over training to enhance cognitive performance. To give participants control over the neurofeedback training, we allowed some of them to freely distribute their training and rest time during the session, whereas others received neurofeedback in an externally paced manner. Importantly, only the distribution of the rest time varied between conditions keeping the rest, training and overall time constant across all participants. To control for non-specific factors, we included a sham-control condition with the pre-recorded feedback from another participant not further involved in this study. We assessed the mental rotation ability, visual search skills, and higher-level cognitive skills task before and after neurofeedback. To estimate the efficacy of neurofeedback we conducted a series of mixed models analyses taking variations between participants into account.

**FIGURE 1:**
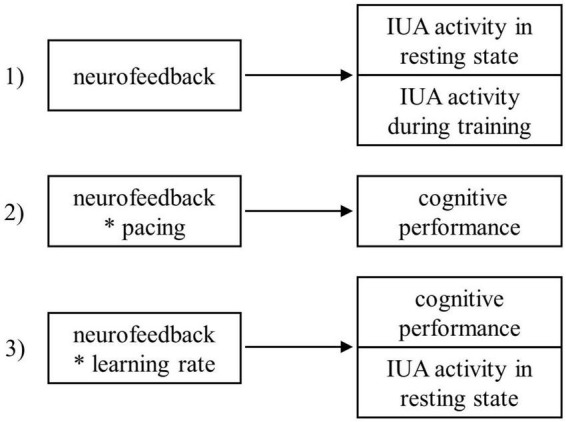
Summary of the hypotheses. The asterisk operator denotes factor crossing.

Based on previous findings, we expected to observe (1) an effect of neurofeedback on relative IUA activity (see section “2.8. Statistical analyses” for more technical details), (2) a transfer effect of neurofeedback and pacing on cognitive outcomes (i.e., a negative effect on changes in error rate and response time), and (3) an association between learning rate during neurofeedback and changes in cognitive outcomes (see Figure 1). We first of all expected to observe a greater increase in IUA activity for participants in the IUA neurofeedback group compared to participants receiving sham neurofeedback after a single neurofeedback session. Additionally, we hypothesized that (2a) all participants increase their cognitive performance from pre to post-neurofeedback due to practice, (2b) participants receiving IUA neurofeedback increase their cognitive performance more than participants receiving sham neurofeedback, (2c) that participants who pace neurofeedback training on their own enhance their cognitive performance more than participants training in an externally paced manner, and (2d) that this pacing effect is more dominant in the real neurofeedback group compared to the sham neurofeedback group. Finally, we expected to observe an effect of neurofeedback learning rates on (3a) cognitive outcomes and (3b) resting-state activity specifically for participants in the real neurofeedback group.

## 2. Materials and methods

This study followed a sham-controlled, randomized, double-blind design to investigate these factors in enhancing both resting-state relative IUA activity and cognitive performance. As we expected larger differences between IUA and sham neurofeedback than between the two pacing conditions within IUA neurofeedback, we required a larger sample size in both IUA neurofeedback pacing conditions to detect smaller effects. Therefore, we randomly allocated participants on a 2:1 basis to either IUA or sham neurofeedback and subsequently on a 1:1 ratio to either self- or externally paced training using the Fisher-Yates algorithm.

### 2.1. Participants

The sample consisted of *N* = 60 healthy young adults (42 females, mean age: 24.65 years, age range: 18–35 years) who underwent the same procedure except for type of neurofeedback and the type of pacing. All participants had normal or corrected-to-normal vision.

### 2.2. Mental rotation task

**FIGURE 2:**
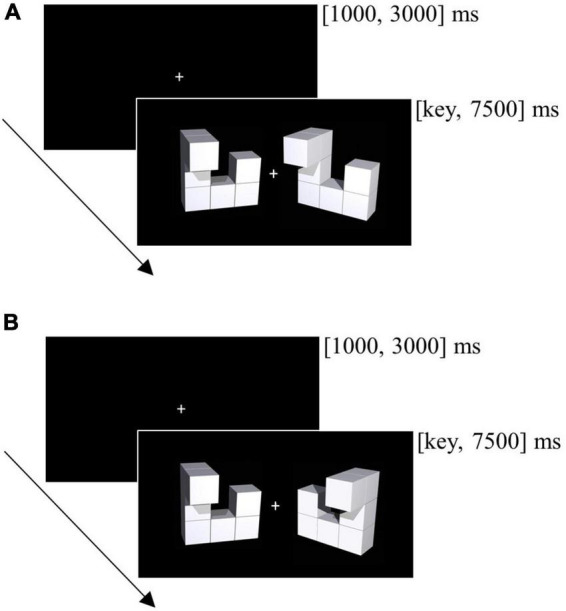
Trial from the mental rotation task. **(A)** The figure presented right to the fixation cross is the same as the figure on the left (correct response is “Y”). **(B)** The figure presented right to the fixation cross is not the same as the figure on the left as it is mirrored (correct response is “N”).

The computerized mental rotation task contained 96 trials and was administered pre- and post-neurofeedback training. Each trial displayed one object pair: a baseline object on the left half of the screen and a target object on the right half of the screen (see Figure 2). The target object was the same as the baseline object (but rotated) for half of the trials and horizontally flipped for the remaining trials. For each participant, time point (pre, post) and rotation angle (0, 50, 100, 150°) we randomly sampled the 3D object pairs without replacement from a stimulus pool (Ganis and Kievit, 2015). Object pairs used for practice trials were excluded from the stimulus pool.

All experimental trials started with a fixation cross. An object pair appeared subsequently until participants responded. If participants did not respond within 7500 ms, the next trial continued. The duration of the fixation cross varied randomly between 1000 and 3000 ms to minimize expectancy effects. Participants were instructed to respond as quickly and as accurately as possible by using the “Y” (same, but rotated objects) and the “N” key (horizontally flipped objects).

### 2.3. Trail making test

The TMT consists of two parts: (A) 25 encircled numbers from “1” to “25” and (B) a total of 25 encircled numbers, from “1” to “13,” and letters, from “A” to “L” (Bowie and Harvey, 2006). We administered a paper pencil version and instructed participants to connect the series of circles in ascending order without lifting the pencil from the paper as fast as they can. For part B participants had to alternate between numbers and letters (i.e., 1-A-2-B etc.). Each part started with a practice trial with a total of eight circles to ensure that participants understood and adhered to the instructions. While the completion time for part A measured visual search and motor speed skills, the completion time for part B assessed higher-level cognitive skills including mental flexibility.

### 2.4. EEG recording

The EEG signals were amplified by a BrainAmp system (Brain Products, Gilching, Germany). Its output was digitized with a resolution of 16 bit and sampled at a rate of 1000 Hz *via* the lab streaming layer protocol. For data acquisition, we mounted a set of 32 Ag/AgCl electrodes according to the 10/20-system and referenced it to FCz. To capture horizontal eye movements, we placed two additional electrodes on the external canthi of both eyes. We kept the impedances below 20 kΩ with the ground and the reference electrode below 5 kΩ throughout the recording.

### 2.5. Neurofeedback

We implemented a neurofeedback software to acquire, process and visualize electrophysiological signals in Python, which will be made available upon reasonable request. Configuration recordings prior to neurofeedback training provided gradient and amplitude artifact detection thresholds calibrated for each participant. A 5-min eyes open resting-state recording prior to the neurofeedback training determined the IUA frequency range (individual alpha peak frequency + 2 Hz) and its related power. We did not disclose successful mental strategies to increase IUA activity to participants before neurofeedback. Some researchers have argued that this reduces the risk of participants enhancing their IUA activity when receiving sham feedback (Naas et al., 2019). Others have pointed out that only the use of mental strategies might increase the targeted brain activity and thus this mental rehearsal should be controlled (Sorger et al., 2019). Nevertheless, the implicitness of some mental strategies complicates the applicability of such a control condition and an explicit instruction might even interfere with the targeted brain activity (Kober et al., 2013). For IUA neurofeedback, we did not find studies experimentally varying the content of mental rehearsal strategies to investigate their efficacy in a randomized controlled trial. For fMRI neurofeedback, studies applying a mental rehearsal control condition typically instructed participants to choose a strategy and to stick to it throughout the session (for a review see Sorger et al., 2019).

During neurofeedback training the IUA band power was averaged across P3, Pz, P4, O1, and O2 (Escolano et al., 2014). The difference of current and resting-state IUA power standardized by the resting-state IUA power standard deviation determined the height of a bar plot. For participants receiving sham feedback we used the same pre-recorded EEG activity. To ensure that the technician carrying out the recording was blinded, he was separated by a mounted wall from the participant and, hence, could not monitor the feedback the participant received. Additionally, the technician only viewed the actual recording presented on the monitor (and not the sham recording). We instructed participants to keep the bar above a line which corresponded to their resting-state IUA power. Whenever the signal exceeded the individually adjusted artifact detection threshold, the word “NOISE” appeared at the center of the screen and the feedback was halted until the signal decreased below the threshold. For online processing, we applied a sliding Fast Fourier Transform performed on a 1 s Hanning window with an overlap of 75% resulting in an update rate of 4 Hz. To increase the frequency resolution to 0.5 Hz, we zero-padded the time windows. To facilitate alpha peak identification and to smooth the spectral data, we additionally applied a Savitzky–Golay filter with window size 11 and polynomial order 2 (Savitzky and Golay, 1964). Participants in the sham condition received the feedback from another participant not involved in the study.

### 2.6. Procedure

**FIGURE 3:**
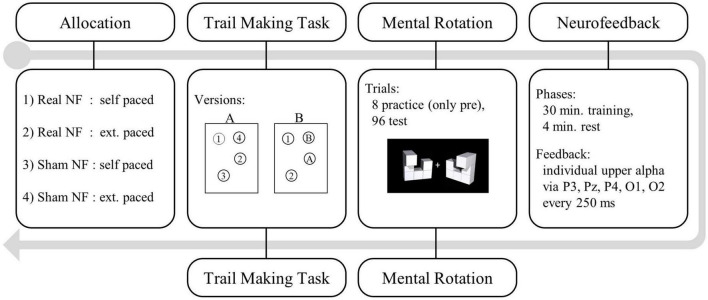
Study procedure: Trial making task (TMT) and mental rotation task were administered pre- and post-neurofeedback.

Participants took part in individual laboratory sessions at the University of Luxembourg. They received course credits or 20€ in vouchers as reimbursement for the 2 h lasting procedure (see Figure 3). After giving their informed, written consent, all participants completed a questionnaire capturing socio-demographic data. We then administered the TMT (pre-neurofeedback) including practice trials and recorded the completion time. Next, we started the EEG recording and continued with the mental rotation task.

To prepare the neurofeedback training, we first calibrated the artifact detection thresholds. Therefore, we recorded three epochs of 10 s each prior to which we instructed participants (1) not to blink, (2) to blink, and (3) to activate other facial muscles. Next, we recorded the baseline IUA activity in resting-state. The single session neurofeedback training procedure had a total duration of 34 min (30 min training + 4 min rest). For sham feedback we provided the same pre-recorded feedback to Participants in the self-paced condition distributed the resting minutes themselves whereas for participants in the externally paced condition five training blocks were interspersed with 1 min resting epochs. When self-pacing the training, resting phases immediately followed upon pressing the spacebar. To continue with the training, we instructed participants in the self-paced condition to press the spacebar again. The bar plot reappeared after a delay of 3 s to reduce the impact of muscle activity on the feedback signal. After the training we administered the post-neurofeedback TMT and mental rotation task without any practice trials. Finally, we debriefed all participants about the allocation of some participants to a control condition receiving sham neurofeedback and about our hypotheses.

### 2.7. EEG pre-processing

We used the MNE library version 0.23.0 (Gramfort et al., 2013) in Python version 3.7.3 (Python Software Foundation, DE, USA) to process all EEG data. For each participant we imported the EEG data into the MNE framework and added all required information (e.g., sampling rate, definition of stimulus channels). We analyzed the data separately for resting states and neurofeedback training. In both cases, we first re-referenced the signal to the average for further analysis. For outlier and artifact detection, we then segmented the continuous EEG data into consecutive, non-overlapping epochs with a duration of 10 s each. Next, we identified and treated outliers either by channel interpolation or by epoch removal. For detection, we estimated for each channel and each epoch the strength of high-frequency components by first calculating the power between 70 and 130 Hz, normalizing it by total power and finally applying a log-transformation for a conversion into decibel. We defined a median absolute deviation threshold of 3 to detect outliers. After raw data inspection, we bandpass filtered the remaining EEG signal between 0.2 and 100 Hz with a finite item response filter (FIR) to improve the following independent component analysis (ICA) performance. In detail, we applied a one-pass, non-causal, zero phase, Hamming-windowed FIR filter (lower half-amplitude cut-off = 0.1 Hz, upper half-amplitude cut-off = 112.5 Hz, passband ripple = 0.0194 dB, stopband attenuation = 53 dB). To attenuate line noise, we additionally notch filtered the signal at 50 Hz. We then performed an ICA decomposition with the FastICA algorithm (Hyvärinen and Oja, 2000) to detect and remove components representing eye- and muscle-artifacts. For artifact detection we relied on the visual inspection of each component’s time signal, its power spectral density distribution and its topographical activity. After the artifact cleaning procedure, we applied the ICA solution back on the EEG data and estimated the power spectral density for each epoch. Only for the resting state phases before and after neurofeedback, we averaged the estimates across all epochs.

### 2.8. Statistical analyses

We carried out all statistical analyses in R version 4.1.3 (R Core Team, 2022). To estimate mixed models, we mostly used the lme4 package version 1.1.28 (Bates et al., 2015) but also used the nlme package version 3.1.155 (Pinheiro et al., 2022) to additionally include autoregressive residuals. For hypothesis testing we performed both model comparisons based on the likelihood ratio test and multiple contrast tests. By default, we determined restricted maximum likelihood estimates of model parameters which produce unbiased parameter estimates and are preferred for unbalanced data (McCulloch and Searle, 2001). Exclusively for model comparisons, we estimated model parameters using maximum likelihood. Since we tested some of our hypotheses with multiple contrast tests, we calculated simultaneous confidence intervals at a 95% significance level with the multcomp package version 1.4.19 which control the family wise error rate (Hothorn et al., 2008).

We fitted a series of mixed models to estimate the fixed effect of time (contrast: post = 0.5, pre = −0.5; continuous for within neurofeedback changes), neurofeedback (IUA = 0.5, sham = −0.5), pacing (self = 0.5, external = −0.5) and their interactions on outcome measures. Depending on the nature of the data we either applied linear mixed models (LMMs) or generalized linear mixed models (GLMMs). For both, we first fitted null models only including random effects to estimate the variance explained by level 1 predictors such as participant or angular disparity (exclusively for the mental rotation task). To calculate point-estimates, we then fitted a full model including all fixed effects; and to estimate the contribution of each fixed effect to the overall model fit, we compared a model including the fixed effect of interest to a corresponding reference model without that effect. Finally, we tested our *a priori* contrasts in the full model including all fixed effects by estimating 95% simultaneous confidence intervals for the specified hypotheses. This procedure corrected the inflated family wise error rate associated with multiple testing. To test our first hypotheses, we evaluated pre vs. post-resting-state and within neurofeedback changes of relative IUA power. For both pre and post-neurofeedback, we calculated the relative IUA power by dividing IUA power by the total power of frequencies up to 49 Hz. Hereby we excluded potential biases due to notch filtering at 50 Hz. For within neurofeedback, we calculated the relative IUA power in the same manner but additionally expressed the change over the 10 s epochs in percentage change from resting-state relative IUA power measured before neurofeedback. To test our second hypotheses, we considered errors and reaction time (only in trials with correct response) for the mental rotation task and completion time for the TMT. For the number of errors, we fitted a GLMM to predict correct and incorrect responses by using a binomial distribution with the logit link function. For both reaction time in the mental rotation task and completion time in the TMT we applied LMMs. Only for the mental rotation task, we additionally included angular disparity as a crossed random effect in the random effects structure to estimate its contribution compared to participants’ characteristics. To test our third hypothesis, we fitted linear models predicting participant level averaged change scores with neurofeedback, pace, learning rate and their interactions. We estimated the learning rate for each participant as the slope in a linear model predicting the change in relative IUA power over neurofeedback training epochs.

## 3. Results

We grouped the results section by the administered tasks and begin with the results on the effect of neurofeedback on IUA in resting state activity and IUA activity during neurofeedback training (hypotheses 1 and 3b). Next, we focus on the behavioral outcomes and report the results for the mental rotation task and the TMT. For each task we report both the crossed effects of neurofeedback and pacing and the crossed effects of neurofeedback and learning rate on performance (hypotheses 2 and 3a). For the descriptive statistics of the mental rotation task and the TMT please refer to Tables 1, 2.

**TABLE 1:** Central tendency and variability of the mental rotation task performance measured as reaction time (RT) in log (ms) for trials with correct response and error rate in ratio of incorrect of trials with any response.

Group	RT		Error rate	
	**Pre-mean (SE)**	**Post-mean (SE)**	**Pre-mean (SE)**	**Post-mean (SE)**
Real, self-paced NF (*n* = 19)	7.90 (0.06)	7.71 (0.06)	16.19 (2.18)	12.14 (2.01)
Real, ext.-paced NF (*n* = 20)	7.89 (0.04)	7.67 (0.04)	13.79 (1.45)	10.34 (1.30)
Sham, self-paced NF (*n* = 10)	7.98 (0.07)	7.80 (0.06)	12.58 (2.26)	9.51 (1.57)
Sham, ext.-paced NF (*n* = 10)	8.00 (0.09)	7.79 (0.09)	16.67 (3.79)	11.39 (3.41)

First, we aggregated the mean of the measures per participant and time point. Then, we aggregated the mean and standard error (SE) of those aggregates. Participants were either allocated to real or sham neurofeedback (NF) and subsequently to either self- or externally paced (ext.-paced) NF. We excluded one participant from the analyses whose overall error rate was above 80%.

**TABLE 2:** Central tendency and variability of the trail making task (TMT) performance measured as completion time in s.

Group	Version A		Version B	
	**Pre-mean (SE)**	**Post-mean (SE)**	**Pre-mean (SE)**	**Post-mean (SE)**
Real, self-paced NF (*n* = 20)	3.20 (0.07)	2.91 (0.07)	3.99 (0.07)	3.74 (0.09)
Real, ext.-paced NF (*n* = 19)	3.07 (0.07)	2.82 (0.05)	3.88 (0.07)	3.77 (0.09)
Sham, self-paced NF (*n* = 10)	3.14 (0.12)	2.92 (0.08)	3.89 (0.06)	3.62 (0.09)
Sham, ext.-paced NF (*n* = 10)	3.16 (0.09)	3.01 (0.09)	3.93 (0.06)	3.87 (0.09)

We aggregated the mean (and standard error) of completion times per TMT version (A,B) group and time point. Participants were either allocated to real or sham neurofeedback (NF) and subsequently to either self- or externally paced (ext.-paced) NF. We excluded one participant from the analyses due to non-compliance with the instructions.

### 3.1. Neurofeedback

First, we estimated the interaction effect of time and neurofeedback condition on resting-state relative IUA power in a linear mixed model. To estimate the effect of learning rate, we then calculated the relative IUA power change score for each participant and included the learning rate in a linear model. For the analysis of the learning rate during neurofeedback training, we finally estimated percentage changes from pre-neurofeedback resting-state relative IUA power over 10 s epochs in a series of linear mixed models.

#### 3.1.1. Resting-state activity

The null model only including a random intercept for participants revealed that participants’ characteristics explained most of the variance (ρ = 0.81) in the relative IUA power measured before and after neurofeedback. A full model including fixed effects for neurofeedback, pacing, time, and their interactions failed to outperform the null model [*X*^2^(7) = 12.04, *p* = 0.1]. This suggests that the incorporated fixed factors did not have sufficient explanatory power and we found no support for changes between pre and post-neurofeedback regarding the relative IUA activity. To estimate the link between learning rate and alterations in resting-state activity, we predicted the difference in relative IUA activity measured during resting-state (post–pre) in a linear model including neurofeedback, pacing, learning rate and their interactions as independent variables. As expected, we observed a positive interaction effect for neurofeedback and learning rate [*b* = 0.18, *t*(52) = 2.10, *p* < 0.05] indicating that the link between learning rate and resting-state relative IUA activity was stronger for participants receiving IUA neurofeedback. A follow-up trend analysis revealed that the more participants increased their IUA activity during neurofeedback, the more they increased their relative IUA activity from pre to post-neurofeedback in the resting-state [*b* = 0.09, *t*(52) = 2.69, *p* < 0.01]. These results indicate that the learning rate plays a crucial role in alternating resting-state activity.

#### 3.1.2. Training activity

To investigate changes during neurofeedback, we estimated parameters of a multilevel growth model including time as a continuous predictor. The time variable represented the ith epoch where each epoch contained 10 s of EEG data during the training phases (the relative IUA power of which we predicted in our models). During pre-processing of the EEG data, we excluded 144 epochs due to artifacts resulting in a total of 10656 remaining epochs. We defined three models (1) a null model with a random intercept for participants, (2) an intermediate model additionally including fixed effects for neurofeedback, pacing, time, and their interaction, and (3) a full model with an additional first order autoregressive, AR(1), within-participant residual. The null model revealed a sufficient intraclass correlation (ρ = 0.28) indicating the adequateness of applying a multilevel model. Our intermediate model outperformed the null model [*X*^2^(7) = 181.31, *p* < 0.001] but out of the three competing models the final model fitted the data best [*X*^2^(1) = 548.33, *p* < 0.001]. As expected, model comparisons to estimate the contribution of each fixed effect revealed a positive main effect for time and a positive interaction effect for time and neurofeedback (see Table 3). After correction for multiple tests by estimating one-sided lower bounds of simultaneous confidence intervals with a 95% family wise confidence level both the main and the interaction effect remained significant. This suggests that although all participants increased their relative IUA power over time, participants in the real neurofeedback group improved their relative IUA power more than participants in the sham neurofeedback group.

**TABLE 3:** Generalized linear mixed model (GLMM) for percentage changes in relative IUA power over neurofeedback training epochs.

Fixed effects					
	***B* (SE)**	**Likelihood ratio test**			**95% SCI-LB**
		*****X***^2^(1)**		***p***	
Intercept	84.191 (3.867)	–		–	–
Neurofeedback	−2.497 (7.734)	0.186		0.67	–
Pacing	−0.367 (7.734)	0.171		0.68	–
Time	0.093 (0.011)	100.441		<0.001	0.070
Neurofeedback × pacing	−18.117 (15.467)	1.292		0.26	–
Neurofeedback × time	0.064 (0.022)	8.557		<0.01	0.017
Pacing × time	0.003 (0.022)	0.059		0.81	–
Neurofeedback × pacing × time	0.011 (0.043)	0.068		0.79	−0.081
**Random effects**					
	**σ^2^**		**phi**		
Participant (intercept)	728.195		–		
Residual	1921.776		–		
Time × participant	–		0.226		

The unstandardized estimates (B) and their standard error (SE) listed for each fixed effect, variance (σ^2^) and auto-correlation (phi) for random effects incorporated in the full model. For completeness, likelihood ratio test result is reported for each fixed effect by comparing a model including the effect to a corresponding reduced model. For final multiple contrast tests, we relied on the lower bound of one sided 95% simultaneous confidence intervals (95% SCI-LB).

A follow-up trend analysis showed that the slope for participants receiving IUA neurofeedback [*b* = 0.13, *t*(10592) = 9.98, *p* < 0.001] was twice as high as the slope for participants receiving sham neurofeedback [*b* = 0.06, *t*(10592) = 3.44, *p* < 0.001]. While participants in the IUA neurofeedback condition increased their relative IUA power by 22.48% over the 180 10 s epochs during training, participants in the sham neurofeedback condition increased their relative IUA power only by 11.02%.

### 3.2. Mental rotation task

We fitted a series of models to estimate the effects of neurofeedback and pacing on changes in mental rotation task performance (i.e., reaction time and errors). From the 11520 trials (192 per participant) we excluded trials from one participant whose overall accuracy during the mental rotation task was below 20% and all trails without a response yielding the remaining 11169 trials. Only for reaction time analysis we additionally excluded trials with incorrect responses after which 9732 trials remained. The central tendency and variability of the outcome measures aggregated over participants and across time (i.e., pre and post) were comparable to results from the validation study (Ganis and Kievit, 2015). As expected, error rates and reaction times increased with angular disparity (see Table 4). To test our hypotheses, we first fitted null models with crossed random intercepts for participant and angular disparity to predict the respective outcome measure. Then, we estimated linear mixed models including the crossed random effects from the null model and the fixed effects for neurofeedback, pacing, time, and their interactions. Finally, we estimated a linear model including individual learning rates during neurofeedback to predict change scores (post–pre) in outcome measures.

**TABLE 4:** Central tendency and variability of mental rotation task performance per angle measured as reaction time (RT) in ms for trials with correct response and error rate in percentage incorrect of trials with response.

Angle	RT	Error rate
	**Mean (SE)**	**Mean (SE)**
0°	2072.03 (72.07)	6.25 (0.99)
50°	2693.48 (87.24)	9.29 (0.92)
100°	3306.65 (94.15)	15.20 (1.32)
150°	3456.22 (95.94)	20.97 (1.46)

We aggregated both measures over participants and across time points (i.e., pre- and post-neurofeedback).

#### 3.2.1. Response errors

The estimation of the null model revealed a similar intraclass correlation for participants’ characteristics (ρ = 0.10) as for the angular disparity of the stimulus itself (ρ = 0.08). Comparisons with the likelihood ratio test estimating the explanatory contribution of each fixed effect revealed a negative main effect for time (see Table 5). Hence, the error rate decreased from pre to post-neurofeedback training independent of the neurofeedback condition participants were assigned to. To test our *a priori* contrasts, we then estimated one-sided 95% simultaneous confidence intervals. The main effect for time remained significant after correcting the inflated family wise error rate. There was no other main effect indicating that the overall error rate was similar across different conditions. Furthermore, the difference in the change of error rate (post vs. pre) between neurofeedback conditions (IUA vs. sham) was negligible. Similarly, we found no three-way interaction effect of time, pacing and neurofeedback.

**TABLE 5:** Generalized linear mixed model (GLMM) for errors in the mental rotation task.

Fixed effects				
	***B* (SE)**	**Likelihood ratio test**		**95% SCI-UB**
		***X*^2^(1)**	***p***	
Intercept	−2.179 (0.296)	–	–	–
Neurofeedback	0.068 (0.189)	0.103	0.75	–
Pacing	−0.049 (0.189)	0	1	–
Time	−0.392 (0.063)	41.808	<0.001	−0.250
Neurofeedback × pacing	0.320 (0.378)	0.760	0.38	–
Neurofeedback × time	0.062 (0.127)	0.271	0.60	0.346
Pacing × time	0.076 (0.127)	0.129	0.72	0.359
Neurofeedback × pacing × time	−0.184 (0.253)	0.517	0.47	0.383
**Random effects**				
	**σ^2^**			
Participant (intercept)	0.413			
Angular disparity (intercept)	0.314			
Residual	3.290			

The unstandardized estimates (B) and their standard error (SE) listed for each fixed effect incorporated in the full model. For completeness, likelihood ratio test result is reported for each fixed effect by comparing a model including the effect to a corresponding reduced model. For final multiple contrast tests, we relied on the lower bound of one sided 95% simultaneous confidence intervals (95% SCI-LB).

To account for individual learning rates during neurofeedback, we additionally estimated a linear model predicting the change scores of error rate with neurofeedback, pacing, learning rate and their interactions as independent variables. Therefore, we aggregated the difference in number of incorrect responses (post–pre) for each participant over all trials. The model showed a positive interaction effect for neurofeedback and learning rate indicating that the linear relationship between error rate and learning rate was more negative for participants receiving IUA neurofeedback than for participants receiving sham neurofeedback [*b* = −17.97, *t*(51) = −1.73, *p* < 0.05]. To investigate whether the linear relationship between the error rate and the learning rate was significantly less than zero for participants in the real neurofeedback group, we performed a follow-up trend analysis. This revealed that the more participants in the IUA neurofeedback condition increased their relative IUA power during neurofeedback the less errors they made in the mental rotation task [*b* = −7.36, *t*(51) = 1.85, *p* < 0.05]. To conclude, the effect of learning rate on reductions in errors differed between the neurofeedback groups with a stronger negative linear relationship for the real than for the sham neurofeedback group.

#### 3.2.2. Reaction time

To reduce the skewness of the distribution, we first log-transformed reaction times. The null model revealed that the variance between participants was greater than the variance between angles. Hence, the logarithmic reaction times depended more on participant’s characteristics than on angular disparity. Like the full model predicting errors, we only found a main effect for time suggesting that all participants reduced their reaction time from pre to post-neurofeedback (see Table 6). However, the groups did not differ in their reduction of reaction time.

**TABLE 6:** Linear mixed model (LMM) for log reaction time in the mental rotation task.

Fixed effects				
	***B* (SE)**	**Likelihood ratio test**		**95% SCI-UB**
		*****X***^2^(1)**	***p***	
Intercept	7.857 (0.134)	–	–	–
Neurofeedback	−0.097 (0.062)	2.525	0.112	–
Pacing	0.014 (0.062)	0.120	0.740	–
Time	−0.204 (0.009)	632.731	<0.001	−0.185
Neurofeedback × pacing	0.031 (0.124)	0.066	0.797	–
Neurofeedback × time	−0.016 (0.017)	0.905	0.341	0.022
Pacing × time	0.030 (0.017)	4.054	0.044	0.068
Neurofeedback × pacing × time	0.016 (0.034)	0.208	0.648	0.092
**Random effects**				
	**σ^2^**			
Participant (intercept)	0.050			
Angular disparity (intercept)	0.068			
Residual	0.159			

The unstandardized estimates (B) and their standard error (SE) listed for each fixed effect incorporated in the full model. For completeness, likelihood ratio test result is reported for each fixed effect by comparing a model including the effect to a corresponding reduced model. For final multiple contrast tests, we relied on the upper bound of one sided 95% simultaneous confidence intervals (95% SCI-UB).

When incorporating learning rates into a linear model to predict change scores of logarithmic reaction time (post–pre) we found a negative interaction effect between neurofeedback and learning rate [*b* = −1373.45, *t*(51) = −1.94, *p* < 0.05]. This suggests that the linear relation between learning rate and changes in reaction time was more negative for IUA neurofeedback. A follow-up trend analysis did not show that the linear relationship was significantly lower than zero for the real neurofeedback group [*b* = −71.35, *t*(51) = −0.26, *p* = 0.40]. In conclusion, the difference in the linear link between learning rate and reaction time was more driven by participants in the sham neurofeedback group who responded slower with increasing learning rate than by participants in the real neurofeedback group who responded faster with increasing learning rate.

### 3.3. Trail making test

To estimate the effects of neurofeedback and pacing on TMT completion time, we fitted a series of models analogously to the analyses of mental rotation task performance. We excluded one participant from our analyses due to non-compliance and ran the models separately for part A and B of the TMT. To reduce the skewness of the completion time distribution, we log-transformed the outcome measure before we fitted the models. First, we fitted a null model with a random intercept for participants. Next, we estimated the full linear mixed models including the fixed effects for neurofeedback, pacing, time, and their interactions.

#### 3.3.1. Part A

The ICC of the null model was high and confirmed the adequateness of a mixed model because 67.7% of the variance in logarithmic completion time was explained by participants’ characteristics. Our model comparisons estimating the contribution of each fixed effect showed a negative main effect for time (see Table 7). This effect remained significant after adjusting for the family wise error. Hence, all participants, irrespective of their group, reduced their logarithmic completion time from pre to post-neurofeedback. The interaction effect of neurofeedback and time as well as the interaction effect of pacing and time were negative but failed to reach significance. When taking the learning rate into account to predict change scores (post–pre) of logarithmic completion time, we did not observe the expected negative interaction between neurofeedback and learning rate. These results suggest that there was no other effect on logarithmic completion time in part A of the TMT except for the practice effect.

**TABLE 7:** Linear mixed model (LMM) for log completion time in part A of the trail making task.

Fixed effects				
	***B* (SE)**	**Likelihood ratio test**		**95% SCI-UB**
		*****X***^2^(1)**	***p***	
Intercept	3.030 (0.038)	–	–	–
Neurofeedback	−0.057 (0.076)	0.578	0.447	–
Pacing	0.025 (0.076)	0.527	0.468	–
Time	−0.229 (0.030)	48.590	<0.001	−0.162
Neurofeedback × pacing	0.165 (0.152)	1.250	0.264	–
Neurofeedback × time	−0.077 (0.059)	1.754	0.185	0.056
Pacing × time	−0.056 (0.059)	0.824	0.364	0.077
Neurofeedback × pacing × time	0.037 (0.119)	0.104	0.747	0.303
**Random effects**				
	**σ^2^**			
Participant (intercept)	0.064			
Residual	0.023			

The unstandardized estimates (B) and their standard error (SE) listed for each fixed effect incorporated in the full model. For completeness, likelihood ratio test result is reported for each fixed effect by comparing a model including the effect to a corresponding reduced model. For final multiple contrast tests, we relied on the upper bound of one sided 95% simultaneous confidence intervals (95% SCI-UB).

#### 3.3.2. Part B

For part B of the TMT the logarithmic completion times varied substantially between participants within the null model (ρ = 0.55). In line with findings for part A, model comparisons showed a negative main effect for time indicating that all participants reduced their logarithmic completion time from pre to post-neurofeedback (see Table 8). Furthermore, the comparisons revealed a negative interaction effect for pacing and time. This suggests that participants in the self-paced group reduced their logarithmic completion time more than participants in the externally paced groups. Both the main effect for time and the negative interaction effect for pacing and time remained significant after adjusting for the family wise error rate with the estimation of simultaneous confidence intervals. Similar to part A, a linear model including the learning rates did not show the expected interaction effect of neurofeedback and learning rate on logarithmic completion time.

**TABLE 8:** Linear mixed model (LMM) for log completion time in part B of the trail making task.

Fixed effects				
	***B* (SE)**	**Likelihood ratio test**		**95% SCI-UB**
		*****X***^2^(1)**	***p***	
Intercept	3.835 (0.039)	–	–	–
Neurofeedback	0.021 (0.079)	0.078	0.780	–
Pacing	−0.051 (0.079)	0.082	0.775	–
Time	−0.170 (0.039)	18.577	<0.001	−0.084
Neurofeedback × pacing	0.187 (0.157)	1.497	0.221	–
Neurofeedback × time	−0.009 (0.077)	0.014	0.905	0.164
Pacing × time	−0.176 (0.077)	5.189	0.023	−0.002
Neurofeedback × pacing × time	0.065 (0.155)	0.188	0.665	0.411
**Random effects**				
	**σ^2^**			
Participant (intercept)	0.062			
Residual	0.040			

The unstandardized estimates (B) and their standard error (SE) listed for each fixed effect incorporated in the full model. For completeness, likelihood ratio test result is reported for each fixed effect by comparing a model including the effect to a corresponding reduced model. For final multiple contrast tests, we relied on the upper bound of one sided 95% simultaneous confidence intervals (95% SCI-UB).

## 4. Discussion

We designed this study to investigate the effects of learning rate and control over training on cognitive performance and electrocortical activity. We randomly assigned participants either to IUA or sham neurofeedback and subsequently to a self- or externally paced single training session using a double-blind design. Before and after neurofeedback all participants performed the mental rotation task and the TMT to measure changes in neurocognitive performance. In line with previous research, we expected that (1) IUA neurofeedback increases IUA activity more than sham neurofeedback. Additionally, we hypothesized that, (2) IUA neurofeedback and self-paced training increase the performance in behavioral tasks to a greater extent than sham neurofeedback and externally paced training. Finally, we expected that, and (3) the neurofeedback learning rate relates to performance increase within the real neurofeedback group. To test these hypotheses, we fitted a series of mixed models and tested the fixed effects with *a priori* contrasts.

We first expected to observe an effect of neurofeedback on relative IUA activity and our analyses demonstrated that participants receiving real neurofeedback increased their IUA activity during training by more than twice as much as participants receiving sham neurofeedback. Regarding resting state IUA activity measured before and after neurofeedback training, we did not find any group differences. This is in line with previous work usually showing differences during training but not in resting-state activity (Escolano et al., 2014). Even studies applying IUA neurofeedback for multiple sessions, did not find changes in resting-state activity between the experimental and a control group (Nan et al., 2012; Navarro Gil et al., 2018). Therefore, it seems unlikely that repeated sessions *per se* would have yielded more pronounced group differences in resting-state activity. More research is needed to specify how changes in brain activity during training relate to behavioral changes measured after the training. One potential explanation is that resting-state alterations depend on the magnitude of IUA power change achieved during neurofeedback (Wan et al., 2014). Our results emphasize the importance of the magnitude of change (i.e., learning rate) to increase the targeted brain activity in resting-state. The more participants increased their IUA activity during neurofeedback, the more they increased their resting-state IUA activity. As an extension of previous findings, we additionally controlled for non-specific factors by comparing this association between the IUA neurofeedback group and a control group receiving sham neurofeedback. This comparison revealed that the positive linear relationship between learning rate and resting-state activity was specific to the real neurofeedback group indicating that participants receiving sham neurofeedback did not increase their IUA activity sufficiently to yield measurable changes when in resting-state. Another potential explanation is that different processes are involved for increased IUA activity through sham feedback compared to real feedback. Nevertheless, future studies applying IUA based neurofeedback should take the learning rate during training as a predictor of differences in resting-state activity into account.

Both the increase in IUA activity from pre-neurofeedback resting-state IUA observed during neurofeedback and during resting-state after neurofeedback are crucial determinants of plasticity induction (Ros et al., 2014). Therefore, we further investigated whether these induced alterations in electrocortical activity transferred to improvements in cognitive performance. Nevertheless, neither for the mental rotation task nor for the TMT were there performance differences between participants receiving IUA neurofeedback and participants receiving sham neurofeedback regarding their improvements from pre to post-training. This finding is in line with the results from another study in which researchers did not observe a significant difference in improvements in mental rotation task performance between those groups after a single session of IUA neurofeedback (Escolano et al., 2014). In contrast to our results, however, the real neurofeedback group decreased their completion time in part B of the TMT more than the sham neurofeedback group. As for changes in electrocortical activity one could argue that a single session of neurofeedback training was not sufficient to yield large improvement differences in cognitive performance between real and sham neurofeedback. In another study with 20 sessions of IUA neurofeedback participants receiving real neurofeedback improved their short-term memory performance more than the control group (Navarro Gil et al., 2018). Importantly, the researchers reported a strong correlation (*r* > 0.5) between the increase in IUA activity from the first to the last session and the increase in short-term memory performance. However, they compared the effects of real neurofeedback to a waitlist control condition which does not rule out non-specific effects and thus might exaggerate the efficacy of IUA neurofeedback (Sorger et al., 2019). We applied a double-blind, sham-controlled design to minimize non-specific effects and our results support the notion of a correlation between the magnitude of change in IUA activity and an increase in cognitive performance. Compared to sham neurofeedback, participants receiving real neurofeedback showed a more positive association between performance gains in the mental rotation task and learning rate. This association, however, was absent in the TMT following the mental rotation task. Compared to the mental rotation task where we administered 96 trials before and after neurofeedback, the TMT consisted of only two parts. Hence, change scores based on the TMT yield less accurate estimates than change scores based on mental rotation task scores which we aggregated for each participant. In summary, our results did not demonstrate an enhanced performance increase under IUA neurofeedback compared to sham neurofeedback, but our results indicate an important role for learning rate for explaining changes in behavioral outcomes.

One of our objectives was to investigate whether self-paced training improved cognitive performance more than externally paced training. As indicated by the positive link between mental strategies and IUA activity as well as the links between self-paced activities and executive functions (Holgado and Sanabria, 2021), we expected to observe increased performance gains for participants self-pacing their training. Our results support this hypothesis demonstrating that self-paced training increased higher-level cognitive skills more than externally paced training. However, the effect of pacing was comparable between IUA and sham neurofeedback indicating that there was no synergistic effect of the combination of IUA neurofeedback and self-paced training. Furthermore, we only found a pacing effect in part B of the TMT which may be explained by the relatively fast completion times in part A implying a ceiling effect. In the mental rotation task, participants who self-paced their training did not improve their performance more than participants receiving externally paced training indicating a task-dependent effect. One conceptual difference between the TMT and the mental rotation task is the degree of self-pacing involved. While participants were not limited in their completion time for the TMT and were allowed to connect the circles at their own pace, we imposed a time limit of 7.5 s per trial in the mental rotation task yielding an externally paced trial procedure if participants did not respond. Furthermore, participants could not influence the presentation duration of the fixation cross throughout the mental rotation task limiting the extent of self-pacing. Future studies are needed to investigate the association of self-pacing involved in both training and cognitive tasks. One way to assess the involvement of conscious and automated processes in self-paced activity would be asking participants to report strategies on how they distributed their rest time during neurofeedback.

Finally, our study has some important limitations regarding the validity of IUA activity as a predictor of resting-state activity and cognitive performance. We designed our neurofeedback procedure to enhance IUA activity during training and based our assumption that this increases cognitive performance on previous studies. Nevertheless, we did not assess whether participants’ IUA activity was a valid indicator of performance in the mental rotation task or the TMT. Furthermore, we did not estimate the change in other frequency bands close to the IUA band, which may have contributed more to the changes in cognitive performance and resting-state activity. Future studies are required to assess the validity of the targeted frequency band to increase a related cognitive function. One approach would be to extract features of neurophysiological signals based on their correlation with cognitive processes to increase the specificity of neurofeedback training and take individual variations into account (Enriquez-Geppert et al., 2017).

## Data availability statement

The raw data supporting the conclusions of this article will be made available by the authors, without undue reservation.

## Ethics statement

The study involving human participants was reviewed and approved by the Ethics Review Panel of the University of Luxembourg (ERP 20-068-A sEEN). The participants provided their written informed consent to participate in this study.

## Author contributions

SU implemented the neurofeedback software, collected the data, performed the data analysis and interpretation, and wrote the original manuscript. CV and SU designed the experiment. CV critically revised the manuscript for the version to be published. Both authors contributed to the article and approved the submitted version.
